# Optimization of Waterborne Poly(Urethane-Acrylate) Nanoemulsions Based on Cationic Polymerizable Macrosurfactants with Different Hydrophobic Side Chain Length

**DOI:** 10.3390/polym11121922

**Published:** 2019-11-21

**Authors:** Guiqiang Fei, Huanqiong Geng, Haihua Wang, Xuan Liu, Yong Liao, Yanming Shao, Mengxi Wang

**Affiliations:** 1 Shaanxi Key Laboratory of Chemical Additives for Industry, Shaanxi University of Science and Technology, Xi’an 710021, China; feiguiqiang@126.com (G.F.); genghuanqiong@126.com (H.G.); liuxuan@sust.edu.cn (X.L.); myquarter@126.com (Y.L.); shaoyanming@sust.edu.cn (Y.S.); jasminecheer@126.com (M.W.); 2 Shaanxi Institute of Technology, College of Chemical Engineering, Xi’an 710300, China

**Keywords:** cationic polymerizable macrosurfactant, waterborne poly(urethane-acrylate), hydrophobic side chain, catalytic chain transfer, mechanism

## Abstract

In situ surfactant-free emulsion polymerization can help avoid the utilization of harmful co-solvents and surfactants in the preparation of waterborne poly(urethane-acrylate) (WPUA) nanoemulsion, but the solid content is extremely limited, which will affect the drying rate and film-forming properties. The utilization of polymerizable macrosurfactants can overcome the above problems. However, the research on cationic polymerizable macrosurfactants is extremely scarce. In this work, cationic dimethylaminoethyl methacrylate-*b*-alkyl methacrylates block copolymers (PDM-*b*-PRMA) with terminal double bonds and different hydrophobic side chain (HSC) lengths were fabricated via catalytic chain transfer polymerization (CCTP). HSC length of PDM-*b*-PRMA played an important role in the phase inversion, morphology, rheological behavior of WPUA nanoemulsions, as well as the comprehensive performance of WPUA/PDM-*b*-PRMA films. Polymerizable PDM-*b*-PBMA macrosurfactant had smaller molecular weight, lower surface tension and colloidal size than the random copolymer (PDM-*co*-PBMA) by traditional free radical polymerization. It was easy for PDM-*b*-PRMA to orientedly assemble at the oil/water interface and provide better emulsifying ability when the carbon number of HSC was four. Compared with WPUA/PDM-*co*-PBMA, WPUA/PDM-*b*-PBMA had a smaller particle size, stability and better film-forming properties. This work elucidated the mechanisms of HSC length in the fabrication of cationic PDM-*b*-PRMA and provides a novel strategy to prepare cationic WPUA of high performance.

## 1. Introduction

Due to its outstanding structure designability, waterborne polyurethane (WPU) has been widely applied in several industrial fields [1,2,3,4,5], like coating, sizing agent, and smart surface material, etc. [6,7,8]. However, WPU also has several deficiencies, including poor water resistance, low thermal stability, and mechanical properties, which limit the extensive utilization of WPU to some extent [9,10]. To enhance its performance, WPU has been modified by crosslinking reactions and hybridization with acrylate, epoxy, organic fluorine and organosilicon [11,12,13,14,15]. Yousefi et al. [16] have designed a robust superhydrophobic and highly oleophobic polyurethane-SiO_2_ nanoparticle coating by using sol-gel process. Lee et al. have [17] developed a poly(urethane-acrylate) (PUA) with enhanced ionic conductivity and excellent mechanical properties.

PUA comprised the advantages of both polyurethane and acrylic polymers, and has been widely used in coatings, adhesives, and surface treatments [17,18]. Up to date, researchers have developed the focus of PUA research into waterborne poly(urethane-acrylate) (WPUA) to reduce the utilization of organic solvents, including anionic, cationic, and nonionic WPUA [19,20]. Heretofore, anionic WPUA has been extensively investigated [21,22,23,24,25]. Fei et al. [26] have used pentaerythritol triacrylate and hydroxyethyl acrylate as cross-linker to fabricate WPUA microemulsions, which was able to increase the mechanical properties and water resistance of cellulose fiber-based paper. Hou et al. [27] have prepared a waterborne anionic alkoxysilane-terminated polyurethane dispersion, which exhibited mechanical properties and excellent freeze-thaw stability.

Compared with anionic WPUA, cationic WPUA displays better adhesion to anionic substrates such as cellulose fiber paper, glass and leather, etc. [28,29,30]. However, investigations on cationic WPUA are relatively rare to date [31,32]. Sheng et al. [33] have obtained WPUA with long-chain hydrophobic alkyl structure by radical polymerization of stearyl acrylate with double bond end-capped WPU, which has been used for water repellents. Heretofore, several approaches have been adopted to prepare WPUA, such as physical blending, chemical blending, seed emulsion polymerization, emulsion copolymerization, mini-emulsion polymerization and in-situ surfactant-free emulsion polymerization [34,35,36,37,38,39]. Co-solvent or surfactant which is harmful to the environment and the product performance is generally utilized during the above polymerization approaches except for in-situ surfactant-free emulsion polymerization. In our previous studies, in-situ surfactant free emulsion polymerization has been employed to fabricate a series of WPUA [40]. For instance, anionic block-copolymers with terminal double-bonds were adopted as polymerizable macrosurfactant to synthesize WPUA nanoemulsions of high solid content and water resistance [41]. Compared to small molecular surfactants, the utilization of polymerizable macrosurfactants can prevent surfactants from migrating onto the surface of film, thereby improving the coating performance [41,42]. However, the investigations on cationic double-bond terminated polymerizable macrosurfactants are scarce, as well as the studies on the utilization of cationic double-bond terminated block-copolymer surfactant for the preparation of cationic WPUA.

In this research, we first report the preparation of cationic dimethylaminoethyl methacrylate-*b*-alkyl methacrylates block copolymers (PDM-*b*-PRMA) with terminal double bonds and different hydrophobic side chain (HSC) length cationic double-bond terminated, as well as its application for the preparation of cationic WPUA colloidal dispersions. The related investigations have been seldom reported, especially for the effects of HSC length. Catalytic chain transfer polymerization (CCTP) was adopted to fabricate cationic double-bond terminated block-copolymer surfmer of controlled molecular weight, which has been demonstrated to be an effective approach to prepare double bond terminated block polymer [43,44]. The morphologies, structures, surface tensions and aggregation behaviors of PDM-*b*-PRMA copolymers were characterized. Effects of HSC length in PDM-*b*-PRMA on the phase inversion behavior and emulsion polymerization of WPUA were also investigated, as well as the film performance.

## 2. Experimental

### 2.1. Materials

Dimethylaminoethyl methacrylate (DM, AR) (Aladdin, Shanghai, China), butyl acrylate (BA, AR) (Aladdin, Shanghai, China), butyl methacrylate (BMA, AR) (Aladdin, Shanghai, China), methyl methacrylate (MMA, AR) (Aladdin, Shanghai, China), ethyl methacrylate (EMA, AR) (Aladdin, Shanghai, China), isooctyl methacrylate (OMA, AR) (Aladdin, Shanghai, China), lauryl methacrylate (LMA, AR) (Aladdin, Shanghai, China), hydroxyethyl methacrylate (HEMA, AR) (Aladdin, Shanghai, China), isopropanol (IPA, AR) (Aladdin, Shanghai, China), azobisisobutyronitrile (AIBN, AR) (Shanpu Chemical Co., Shanghai, China), methyl ethyl ketone (MEK, AR) (Aladdin, Shanghai, China), azodiisopropyl imidazoline (AIBI, AR) (Runxing Photoelectric Material Co., Qingdao, China), [bis-(aqua) bis-(difluoroboryl) dimethylglyoximato] cobalt(II) (CoBF, 99%) (Janus New Materials Co., Xi’an, China) and glacial acetic acid (HAc, AR) (Aladdin, Shanghai, China) were applied to be main materials. Isophorone diisocyanate (IPDI, Technical Grade) (Degussa, Frankfurt, Germany), polycaprolactone diol (PCL1000) (Degussa, Frankfurt, Germany), methyldiethanolamine (MDEA, AR) (Aladdin, Shanghai, China) and trimethylolpropane (TMP, AR) (Kermel, Tianjin, China) were utilized after vacuum-dehydration. DM, MMA, EMA, BMA, OMA, LMA, AIBN and MEK were used after purification.

### 2.2. Preparation of PDM-b-PRMA Macrosurfactants through the CCTP Process

A mixture of MEK (30 g), AIBN (0.08 g), and CoBF (3.5 mg) in a 250 mL three-necked round-bottomed flask was degassed by bubbling with nitrogen. The flask was submerged in a water bath and stirred at 75 °C. Then, 15.7 g DM and 2 mg CoBF were injected into the constant pressure funnel within 1 h. After another 2 h, the flask was immersed in ice-water bath immediately to quench the polymerization, then PDM was obtained after removing MEK with vacuum evaporation.

**Figure 1:**
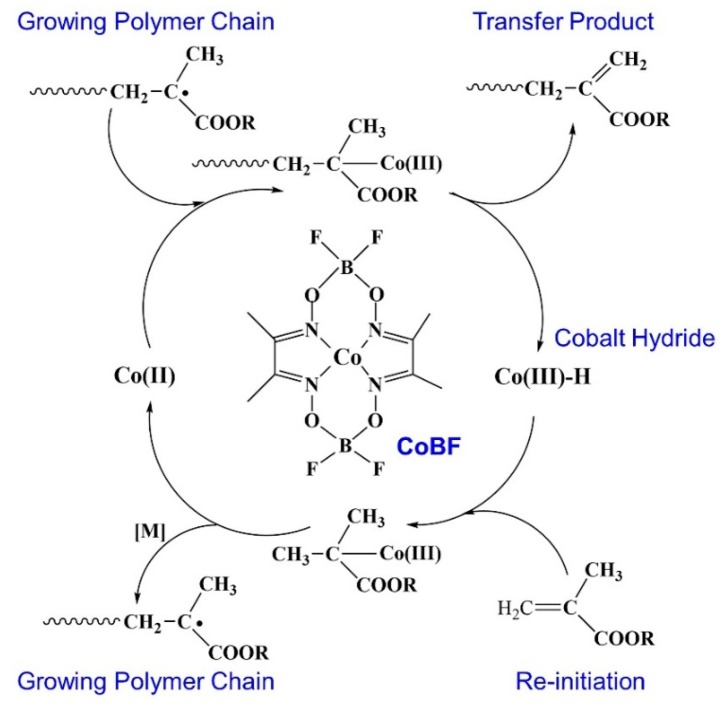
The synthetic process of catalytic chain transfer polymerization (CCTP).

A mixed of PDM, MEK, IPA, and AIBN in a 250 mL three-necked round-bottomed flask was degassed by bubbling with nitrogen. Then, RMA and CoBF were injected into flask after deoxygenation. The flask was immersed in a water bath and magnetically stirred at 75 °C for 5 h. After polymerization, the macrosurfactants were obtained by using the precipitation/dissolution method. The formulations of PDM-*b*-PRMA with different HSC lengths are shown in Table 1. The synthetic process of CCTP was shown in Figure 1. Additionally, PDM-*co*-PRMA and PDM(r) were prepared as control samples by free radical polymerization conventionally.

### 2.3. Preparation of WPUA Emulsion Based on PDM-b-PRMA Macrosurfactants

PCL1000 (10 g), IPDI (7 g), MDEA (1.74 g) and TMP (0.15 g) were placed in a three-necked round-bottomed flask under continuous stirring, BA (4 g) and MMA (12 g) were then placed in it and performed the degree at 75 °C. After 2 h reaction, the temperature was raised to 80 °C and HEMA (1.42 g) was added. The reaction was operated for another 30 min. Then, the reaction system was naturally cooled down to 40 °C and the metered glacial acetic acid was added within 30 min to obtain a double bond terminated PU prepolymer. PDM-*b*-PRMA (1.09 g) was then slowly added into the PU prepolymer under vigorous stirring. After emulsification for 30 min, a stable translucent cationic PU pre-emulsion with free MMA and BA monomers was obtained. Then AIBI initiator was introduced dropwise in 2 h at 75 °C to start the radical polymerization. After another 2 h reaction, the WPUA emulsions modified with PDM-*b*-PRMA were prepared. The synthesized emulsions were recorded as WPUA/PDM-*b*-PRMA. WPUA/PDM-*b*-PRMA emulsions. 30 g emulsion was then poured onto PTFE plates, and dried at room temperature for seven days and then 75 °C for 12 h to form WPUA/PDM-*b*-PRMA latex films. WPUA/PDM-*co*-PBMA was also prepared for comparison.

**Figure 2:**
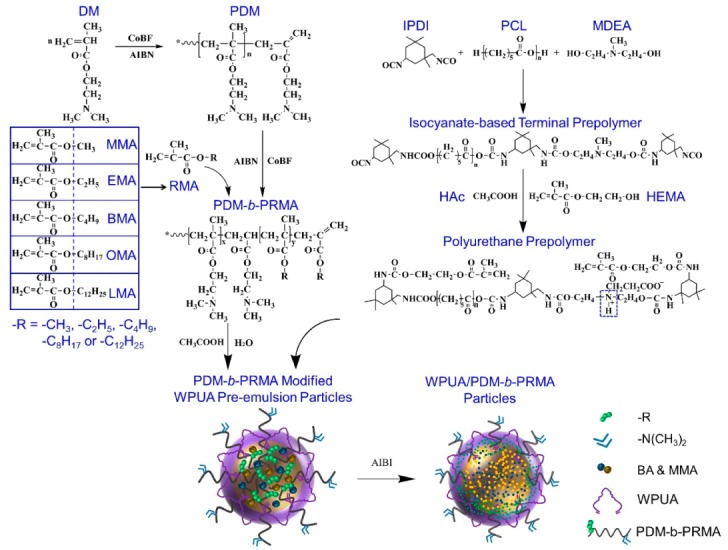
Schematic preparation of PDM-*b*-PRMA and WPUA/PDM-*b*-PRMA emulsions.

The molecular structures and preparation scheme of PDM-*b*-PRMA and WPUA/PDM-*b*-PRMA emulsions are shown in Figure 2.

### 2.4. Characterizations

FTIR spectra were performed by Fourier transform infrared spectrometer (FTIR, Vector-22) Bruker, Karlsruhe, Germany). The scan ranged from 4000 to 500 cm^−1^. ^1^H-NMR spectra were obtained with nuclear magnetic resonance spectrometer (NMR, Avance 400 MHz) (Bruker, Karlsruhe, Germany). Molecular weight was determined on a gel permeation chromatograph (GPC, Waters 2695) (Waters, Milford, Massachusetts, America) with polystyrene as a standard. The sample was diluted to 3 mg/mL, and the sample dosage was 50 μL. The test was conducted at 40 °C and the flow rate was 0.8 mL/min. The iodine value of the macrosurfactants was measured as follows. The PDM-*b*-PRMA sample was dissolved in distilled water, and then a certain amount of potassium bromate-potassium bromide (KBrO_3_-KBr) solution and 2 mL hydrochloric acid were added. The mixture was sealed and kept in the dark area for 2 h, afterwards a quantitative potassium iodide (KI) solution was added and kept in the dark for another 5 min. Lastly, sodium thiosulfate (Na_2_S_2_O_3_) was used as titration reagent of the solution. When the solution was pale yellow, 1 mL of starch solution (0.1 g/L) was added. Titration was continued by adding Na_2_S_2_O_3_ to the solution until the blue color disappeared. The volume of the Na_2_S_2_O_3_ solution was *V*. Then, to make a blank control, the volume of Na_2_S_2_O_3_ solution used was *V*_0_. The iodine value (*X*) of samples was calculated according to Equation (1):

(1) $$X=\frac{C_{0}(V_{0}-V)\times 0.1269\times 100}{M}$$

where *C*_0_ is the solution concentration of Na_2_S_2_O_3_ and *M* is the weight of PDM-*b*-PRMA.

Thermogravimetric analysis was carried out with a thermogravimetric analyzer (TG, TGA Q500) (TA, Newcastle, Delaware, America), heating at a rate of 10 °C/min at a temperature ranging from 20 to 600 °C under N_2_ atmosphere. Surface tension was measured on a tensiometer (DCAT21) (Dataphysics, Stuttgart, Germany). The micelle aggregation behavior was investigated with fluorescence emission spectroscopy which used pyrene as a probe. The particle size of emulsion was determined on Zetasizer Nano-ZS90 particle size analyzer (Malvern, Malvern, England). Morphology was observed on a transmission electron microscope (TEM, Tecnai G2 F20) (Hitachi, Tokyo, Japan).

The relationship between the storage modulus (G′) (G’), the loss modulus (G″), and the loss tangent (tan *δ*) of the emulsions with oscillation frequency were measured by a rheometer (AR2000ex) (TA, Newcastle, Delaware, America). The temperature was 25 °C, frequency sweep ranged from 0.1 to 100 Hz and fixed strain was 20%. The latex films were carefully peeled off from the glass and tested by a total reflection infrared (ATR-FTIR, VECTOR-22) (Bruker, Karlsruhe, Germany). The films were immersed in water for 24 h firstly, and then observed by atomic force probe scanning electron microscope (AFM, SPI3800-SPA-400) (Seiko, Tokyo, Japan) at a test temperature of 25 °C and relative humidity of 33.5%. The solid content of emulsions was tested according to GB-1725-79. The contact angle (*θ*) of water adhering to the latex film surface was measured using a contact angle meter JC2000A (Zhongchen Digital Technology Equipment Co., Shanghai, China). The mechanical properties of the latex films were carried out with a universal testing machine XWW-20B (Jiande Testing Instrument Co., Chengde, China) while the stretching speed was 500 mm/min.

## 3. Results and Discussion

### 3.1. Structural Analysis

**Figure 3:**
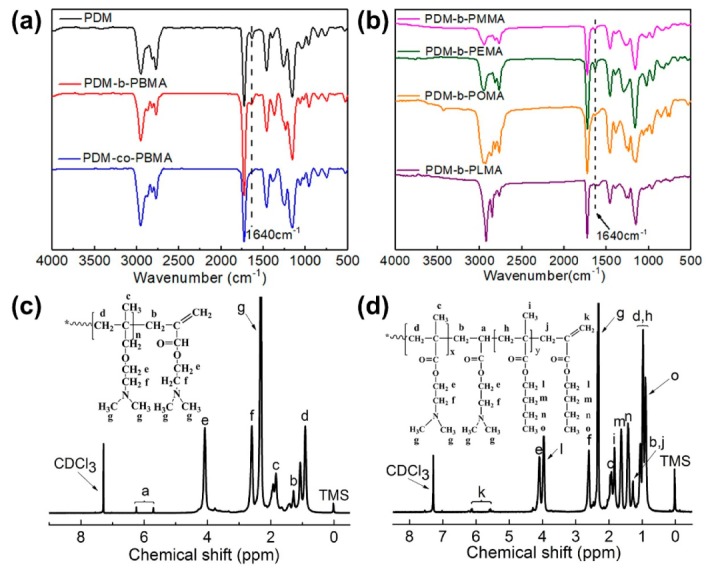
FTIR spectra of PDM, PDM-*b*-PBMA, PDM-*co*-PBMA (**a**) and PDM-*b*-PRMA (**b**), ^1^H-NMR spectra of PDM (**c**) and PDM-*b*-PBMA (**d**).

The structure of PDM-*b*-PRMA was characterized with FTIR, ^1^H-NMR and GPC. As shown in Figure 3a,b, the characteristic absorption peak located at 1640 cm^−1^ can due to terminal -C=C of PDM-*b*-PRMA [45], there was no -C=C characteristic absorption peak existing in PDM-*co*-PBMA, which suggested that surfactants prepared by traditional free radical polymerization had no polymerizable double bond.

In the ^1^H-NMR spectrum of PDM, the characteristic peaks of the vinyl group appeared at 6.12 and 5.65 ppm (Figure 3c), indicating that PDM obtained by the CCTP process possessed terminal -C=C groups. In the ^1^H-NMR spectrum of PDM-*b*-PBMA (Figure 3d), the characteristic peaks corresponding to -C=C groups were also detected at 5.5 and 6.2 ppm, certifying the presence of -C=C groups in PDM-*b*-PBMA. The peak at 4.05 ppm could be ascribed to -O-CH_2_- in DM unit and the peak at 3.94 ppm is due to the hydrogen of -O-CH_2_- in the BMA unit, proving that BMA monomer was successfully polymerized into the chain of the PDM.

**Figure 4:**
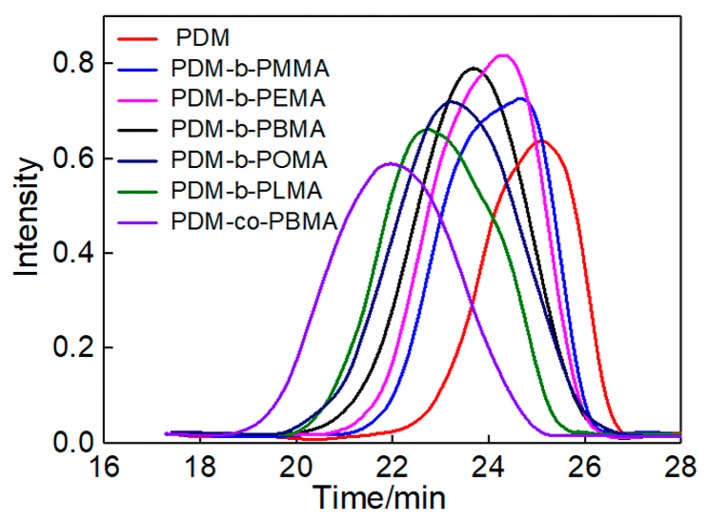
Effect of hydrophobic side chain (HSC) length on the molecular weight of PDM-*b*-PRMA.

The molecular weight and polydispersibility (PDI) of PDM, PDM-*b*-PRMA and PDM-*co*-PBMA measured by GPC were shown in Figure 4 and Table 2. The molecular weight and PDI of PDM-*co*-PBMA were significantly larger than that of PDM-*b*-PRMA, suggesting that the CCTP method was able to reduce the molecular weight and PDI of the copolymer. The iodine value (*X*) of PDM-*b*-PRMA and PDM-*co*-PBMA was also measured to evaluate the degree of unsaturation based on our previous study [40].

*M_n_* and *M_w_* were measured by GPC. *M_n_*′ was measured by the iodometric method and estimated by Equation (2):

(2) $${M_{n}}^{\prime }=\frac{12690\times 2}{X}$$

PDI is the polydispersibility index and *D* is the polymerization degree of PRMA. *C* is the content of PDM-*b*-PRMA with terminal C=C, which was estimated by Equation (3):

(3) $$C=\frac{M_{n}}{{M_{n}}^{\prime }}\times 100\%$$

With the increasing of HSC length of RMA, the molecular weight and PDI of PDM-*b*-PRMA copolymers increased, while the proportion between PDM-*b*-PRMA molecules containing double bonds and PDM-*b*-PRMA molecules without double bonds decreased. With increasing the HSC length, the chain entanglements were enhanced. On the one hand, the free radicals were wrapped with entangled polymer chains, thereby reducing the reactivity between macromolecular chain radicals and Co(II) and decreasing the catalytic chain transfer constant, resulting in the increase of molecular weight. On the other hand, the probability of conversion from Co(II) to Co(III)-H decreased with the increasing of entanglement of free radical chains and polymers chain, i.e., the catalytic efficiency decreased, leading to the increase in the formation of copolymer without terminal double bonds, as well as the molecular weight and PDI. Furthermore, it was also reported in the literature that the glass transition temperature significantly decreased with increasing the aliphatic side chain size, indicating internal plasticization and an easing of the movement of chain segment [46], which is beneficial for the polymerization of RMA and thereby increase the polymerization degree (D).

### 3.2. Thermal Behavior

**Figure 5:**
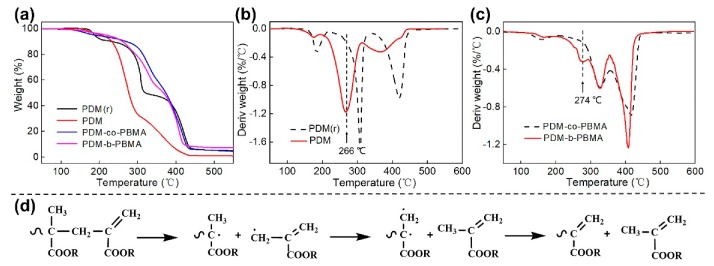
Thermogravimetric (TG) curves of PDM(r), PDM, PDM-*co*-PBMA and PDM-*b*-PBMA (**a**), derivative thermogravimetry (DTG) curves of PDM(r) and PDM (**b**), DTG curves of PDM-*co*-PBMA and PDM-*b*-PBMA (**c**), decomposition mechanism of PDM-*b*-PBMA with terminal double bond (**d**).

Thermal behaviors of PDM and PDM-b-PBMA were investigated with TG and derivative thermogravimetry (DTG), as presented in Figure 5. The decomposition temperature at 5% weight loss for PDM and PDM(r) was found to be 204 and 187 °C, respectively. It indicated that the PDM was endowed with higher thermal stability (Figure 5a). And their thermal decomposition could be divided into three stages (Figure 5b). The first decomposition stage at 170 °C was corresponding to the decomposition of the head-to-head structure generated by coupling termination. The weight loss of PDM (r) (0.338%) was higher than that of PDM (0.121%) since PDM with terminal double bonds was formed during the CCTP process, which prominently reduced the probability of coupling termination. The second decomposition stage for PDM appeared at 215–310 °C was ascribed to the characteristic decomposition of terminal double bonds, the related decomposition mechanism was presented in Figure 5d [40,47], which showed a decomposition possibility of terminal double bond of PDM-*b*-PRMA, leading a cycle with Co(II) and Co(III)-H. The third stage at 371 °C was assigned to the decomposition of polymer backbones [48]. In contrast, no characteristic peak for the decomposition of terminal double bonds was observed for PDM(r), and two decomposition peaks at 310 and 420 °C were detected, which can be due to the decomposition of side chains and main chains.

The thermal stability of PDM-*b*-PBMA and PDM was also significantly lower than that of PDM-*co*-PBMA and PDM(r) (Figure 5a), which can be assigned to the decreased molecular weight. Additionally, PDM-*co*-PBMA displayed three decomposition stages, while PDM-*b*-PBMA presented four decomposition stages. A characteristic peak corresponding to the decomposition of terminal double bonds was found at 274 °C [40,47], further certifying the formation of double bonds at the end of PDM-*b*-PBMA chains.

### 3.3. Solution Property Analysis

**Figure 6:**
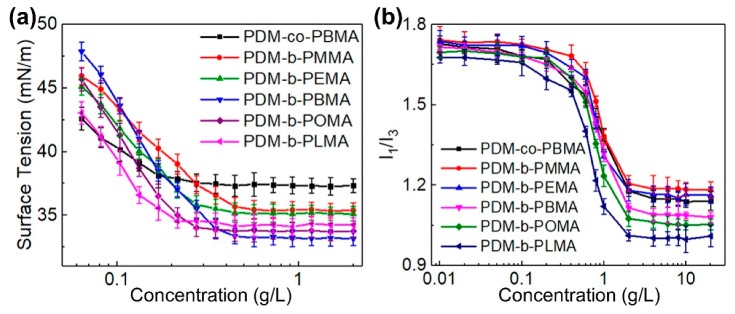
Effect of HSC length on the surface tension of PDM-*b*-PRMA (**a**). Relationship between PDM-*b*-PRMA concentration and I_1_/1_3_ (**b**).

The surface tension of PDM-*b*-PRMA in aqueous solution was determined, as shown in Figure 6a. Some related parameters, including critical micelle concentration (*CMC*), surface tension, surface excess concentration (*Γ*) and minimum surface area per molecule (*A*) were calculated based on our previous study [40] and summarized in Table 3. In comparison with PDM-*co*-PBMA, the surface tension of PDM-*b*-PRMA decreased from 37.36 mN/m to 33.27 mN/m. Since PDM-*co*-PBMA chains with irregular structure tended to lie parallelly at the air/water interface, leading to the increase of *A* and the decrease of *Γ*, the surface tension was thereby increased.

With increasing the length of HSC, the surface tension decreased to 33.27 mN/m for PDM-*b*-PBMA. When the length of HSC was short, the *Γ* at air/water interface decreased owing to the increased static repulsion induced by a denser distribution of positive charge, resulting in the increase of surface tension. Moreover, short HSC length was easily hydrated with water molecules, the migration ability from water to air/water interface was thereby weakened, leading to a decrease of *Γ*. On the contrary, the migration ability and *Γ* increased with increasing the length of HSC, and lower surface tension was achieved. However, when OMA and LMA were adopted, the surface tension increased because of the high steric hindrance.

Micelle aggregation behavior was also investigated by the fluorescence emission spectrum with pyrene. The peak intensity ratio (I_1_/I_3_) of the first vibronic peak to the third of pyrene was tested at different concentrations for PDM-*co*-PBMA and PDM-*b*-PRMA. When the macrosurfactant concentration under *CMC*, pyrene firstly located in polar environment and then migrated into an environment of increased hydrophobicity as the concentration was higher than *CMC*, leading to an abrupt decrease of I_1_/I_3_. Afterwards, I_1_/I_3_ leveled off when all of the pyrene entered into the micelles. It was found that I_1_/I_3_ decreased with increasing the length of HSC, indicating more pyrene molecules entered into the micelles due to the more hydrophobic environment.

**Figure 7:**
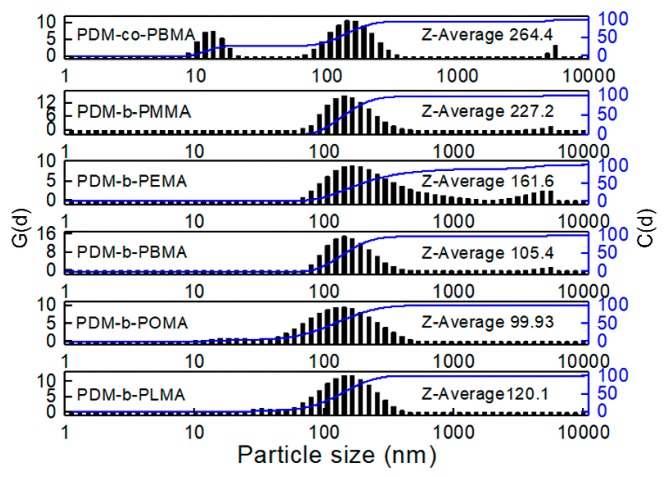
Particle size and distribution of PDM-*co*-PBMA and PDM-*b*-PRMA with different HSC lengths.

The average colloidal particle size of PDM-*co*-PBMA and PDM-*b*-PRMA micelles were investigated, as illustrated in Figure 7. The particle size of PDM-*co*-PBMA was 264.4 nm, and the distribution displayed as bimodal distribution. The particle size decreased to 105.4 nm for PDM-*b*-PBMA. The particle size kept decreasing to 99.93 nm for PDM-*b*-POMA and then increased to 120.1 nm. It indicated that enhanced hydrophobic association and interactions in PDM-*b*-PRMA were favorable to decrease the particle size.

### 3.4. Phase Inversion Analysis of PUA Prepolymer and Application of Cationic Macrosurfactants to WPUA Nanoemulsions

**Figure 8:**
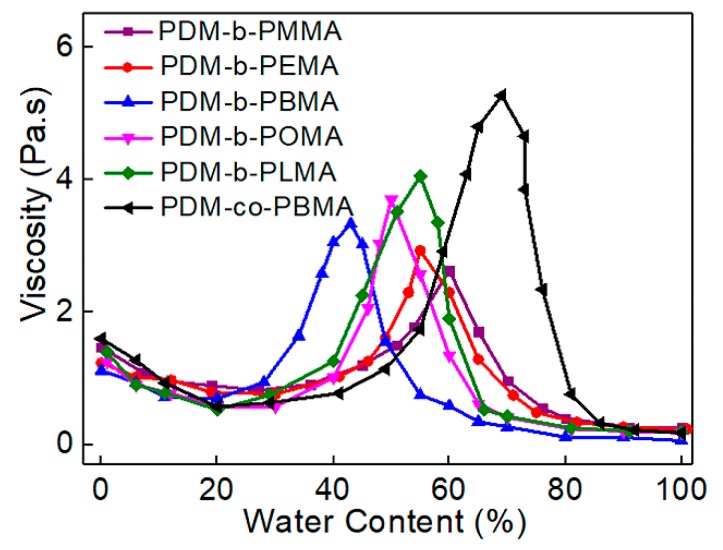
Influence of HSC length on the viscosity of PUA prepolymers during phase inversion process.

PDM-*co*-PBMA and PDM-*b*-PRMA macrosurfactants were utilized with water to assist the phase inversion process of poly(urethane-acrylate) prepolymer. The function of PUA prepolymer viscosity with water content is presented in Figure 8. The water content at phase inversion point (PIP) for PDM-*co*-PBMA was 68%, resulting in the decrease of WPUA solid content, which was detrimental to the drying rate of waterborne coatings (Table 4). The viscosity of PUA prepolymer emulsified with PDM-*co*-PBMA was higher compared with PUA prepolymer emulsified with PDM-*co*-PBMA. It can be ascribed to the increased chain entanglements induced by the random chain structures.

The water content at PIP decreased to 43% when PDM-*b*-PBMA was utilized, and the WPUA solid content increased to 40%. However, the water content at PIP increased from 43% to 55% with further increasing the carbon number of HSC from 4 to 12, leading to a decrease of maximum solid content. The length of HSC played an important role in the PUA prepolymer phase inversion process and the maximum solid content of cationic WPUA nanoemulsions.

**Figure 9:**
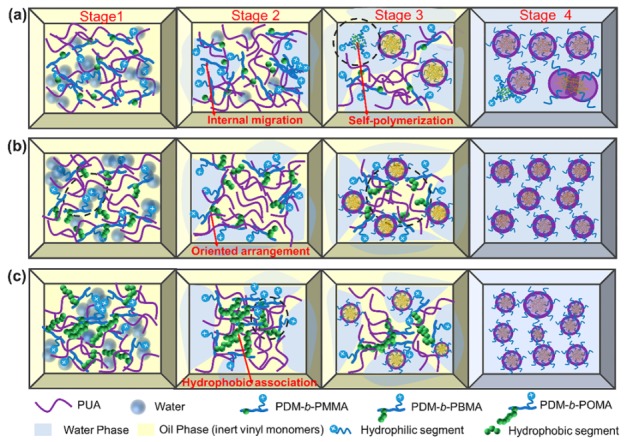
Schematic models of macrosurfactants PDM-*b*-PMMA (**a**), PDM-*b*-PBMA (**b**) and PDM-*b*-POMA (**c**) application in the phase inversion process of poly(urethane-acrylate) prepolymer at different stages.

The phase inversion mechanisms of PUA prepolymer and formation of WPUA nanoemulsions with the addition of PDM-*b*-PRMA of different HSC lengths were summarized in Figure 9. The phase inversion and polymerization process can be divided into four stages. At stage 1, polymerizable PDM-*b*-PRMA macrosurfactant and water were introduced into the PUA prepolymer to emulsify the PUA prepolymer. The interactions among PUA chains were weakened due to the static repulsion between cationic PUA and cationic PDM-*b*-PRMA, leading to a slight decrease in viscosity. At stage 2, the solvation sheath of HSC was weakened and associated with each other, resulting in an increase of viscosity. At stage 3, the interpolymeric networks were associated with further increasing the water content, and O/W micelles were thereby generated. The viscosity was simultaneously decreased. At stage 4, the viscosity kept decreasing owing to the formation of micelles, and then leveled off when the phase inversion was completed.

With increasing the length of HSC, the viscosity increased because of the enhanced hydrophobic association. In addition, it is easier for PDM-*b*-PBMA macrosurfactant to distribute at the oil/water interface due to its low surface tension and moderate flexibility, resulting in a decrease of water content at PIP. The water content at PIP for PDM-*b*-PMMA increased since the migration of PDM-*b*-PMMA to the interface can be restricted due to its high hydrophilicity and high surface tension (Figure 9b). The water content at PIP for PDM-*b*-POMA increased since the migration of PDM-*b*-PMMA to the interface can be restricted due to enhanced chain entanglements induced by longer side chains and high surface tension (Figure 9c).

**Figure 10:**
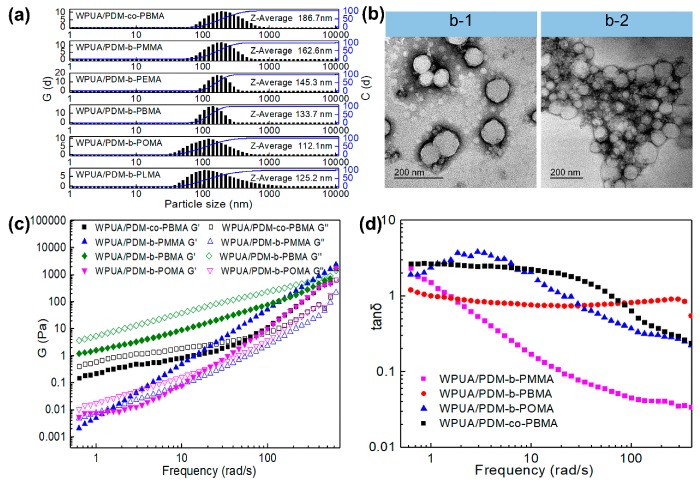
Effects of PDM-*b*-PRMA and PDM-*co*-PBMA on micelle size of WPUA/PDM-b-PRMA and WPUA/PDM-*co*-PBMA nanoemulsions (**a**). TEM images (**b**) of WPUA/PDM-*b*-PBMA (**b-1**) and WPUA/PDM-*co*-PBMA (**b-2**). Effect of PDM-*b*-PRMA and PDM-*co*-PBMA on storage modulus and loss modulus of nanoemulsions (**c**). Effect of PDM-*b*-PRMA and PDM-*co*-PBMA on loss angle tan δ of nanoemulsions (**d**).

Particle size, morphology and rheology of WPUA/PDM-*co*-PBMA and WPUA/PDM-*b*-PRMA nanoemulsions were also investigated, as shown in Figure 10. The particle size was 186.7 nm for WPUA/PDM-*co*-PBMA nanoemulsion while the particle size of WPUA/PDM-*b*-PBMA nanoemulsion decreased to 133.7 nm (Figure 10a). More nucleation sites were generated when PDM-*b*-PBMA macrosurfactant with higher activity was utilized, resulting in the formation of micelles with smaller particle size and narrower distribution. The TEM morphology of WPUA/PDM-*co*-PBMA colloidal particles also displayed wide particle size distribution, and the colloidal particles adhered to each other. In contrast, the WPUA/PDM-*b*-PBMA colloidal particles were more uniform and dispersed with each other (Figure 10b).

The particle size decreased with increasing the length of HSC, and then increased for WPUA/PDM-*b*-PLMA. The decreased particle size can be due to the lower surface activity, as well as enhanced hydrophobic association and interactions inside micelles. However, the solubility of PDM-*b*-PLMA decreased because of the longer HSC, leading to the formation of larger micelles.

The rheological behaviors of WPUA/PDM-*co*-PBMA, WPUA/PDM-*b*-PMMA, WPUA/PDM-*b*-PBMA, and WPUA/PDM-*b*-POMA nanoemulsions showed that the storage modulus (G’) of WPUA/PDM-*co*-PBMA nanoemulsion was lower than that of WPUA/PDM-*b*-PBMA nanoemulsion. It suggested that the interactions among WPUA/PDM-*co*-PBMA colloidal particles were higher than those of WPUA/PDM-*b*-PBMA colloidal particles. Additionally, G’ of WPUA/PDM-*b*-PBMA increased with increasing the frequency, while G’ of WPUA/PDM-*co*-PBMA increased slightly with frequency, indicating stronger networks among WPUA/PDM-*co*-PBMA colloidal particles which were difficult to destroy under external force.

It can be also observed that G’ of WPUA/PDM-*b*-PBMA nanoemulsion was the highest, G’ of WPUA/PDM-*b*-POMA nanoemulsion was the lowest, and G’ of WPUA/PDM-*b*-PMMA was in between. Notably, G’ of WPUA/PDM-*b*-PMMA was higher than G″ of WPUA/PDM-*b*-PMMA, indicating that WPUA/PDM-*b*-PMMA exhibited elastic behavior. In contrast, G” was higher than G’ for WPUA/PDM-*b*-PBMA and WPUA/PDM-*b*-POMA nanoemulsions, presenting viscous behavior. Moreover, the loss angle tan *δ* of WPUA/PDM-*b*-PBMA nanoemulsion kept almost invariable at 1, certifying that the WPUA/PDM-*b*-PBMA nanoemulsion was endowed with the optimum stability.

### 3.5. Water Resistance, Thermal Stability, and Mechanical Properties Analysis

**Figure 11:**
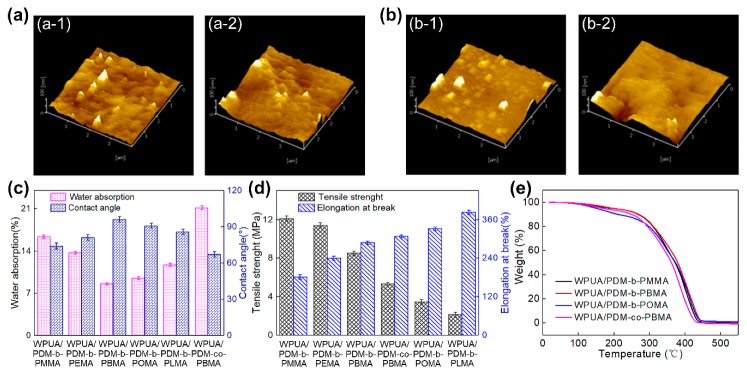
AFM images of WPUA/PDM-*b*-PBMA latex film immersed in water for 0 h (**a-1**) and 24 h (**a-2**), WPUA/PDM-*co*-PBMA latex films immersed in water for 0 h (**b-1**) and 24 h (**b-2**). Effect of PDM-*b*-PRMA and PDM-*co*-PBMA on the water absorption (**c**), mechanical properties (**d**) and thermal stability (**e**) of WPUA/PDM-*b*-PRMA and WPUA/PDM-*co*-PBMA latex film.

AFM were utilized to investigate the structure and morphology variation before and after immersion in water for 24 h (Figure 11a,b). For WPUA/PDM-*b*-PBMA membrane, little changes took place in the surface morphology, while the WPUA/PDM-*co*-PBMA membrane had significant changes. As shown in Table 5, in comparison with WPUA/PDM-*co*-PBMA film, the surface roughness of WPUA/PDM-*b*-PBMA film just had slightly changes because the structure of PDM-*b*-PBMA owned -C=C which can be polymerized with vinyl monomers in the colloidal particles. There was only physical adsorption between PDM-*co*-PBMA and WPUA particles. It is easy for PDM-*co*-PBMA to migrate to the surface of the WPUA/PDM-*co*-PBMA membrane, resulting in significant changes in the surface roughness of WPUA/PDM-*co*-PBMA latex film. The phenomena proved that WPUA/PDM-*b*-PBMA membrane was endowed with improved water resistance compared with WPUA/PDM-*co*-PBMA membrane.

Water absorption results also showed that the water absorption of WPUA/PDM-*co*-PBMA membrane was 21.2% (Figure 11c). The water absorption of WPUA/PDM-*b*-PBMA membrane decreased to 7.3%, and the contact angle increased from 74.2° to 96.3°, which was consistent with the above FTIR and AFM results. It was difficult for PDM-*b*-PBMA macrosurfactant to migrate to the membrane surface owing to the formation of covalent bonds between PDM-*b*-PBMA and WPUA, resulting in the increased water resistance. Mechanical properties displayed that the tensile strength of WPUA/PDM-*co*-PBMA and WPUA/PDM-*b*-PBMA membranes were found to be 5.3 and 8.5 MPa (Figure 11d), certifying that the interactions between PDM-*b*-PBMA and WPUA were enhanced.

With increasing the length of HSC, the tensile strength had a significant decrease, ranging from 12.1 to 2.2 MPa and the elongation at break increased from 181% to 383%. It demonstrated that the interactions were weakened when longer HSC were adopted, leading to a decrease of tensile strength. However, the elongation at break increased with the increase of side-chain flexibility. Simultaneously, the water absorption decreased from 16.4% to 8.6% and then increased to 11.4%, the contact angle increased from 74.2° to 96.3° and then decreased to 86°. Thermal behaviors for WPUA/PDM-*b*-PRMA and WPUA/PDM-*co*-PBMA were also analyzed, as shown in Figure 11e. The decomposition temperature at 5% weight loss for WPUA/PDM-*b*-PBMA was 196.4 °C and 170.9 °C for WPUA/PDM-co-PBMA. It was also found that the decomposition temperatures at 5% weight loss for WPUA/PDM-b-PMMA and WPUA/PDM-b-POMA were 195.6 °C and 152.4 °C. Thermal stability analysis certified that WPUA/PDM-b-PBMA membrane possessed relatively better thermal stability. In conclusion, the water resistance, mechanical properties, and thermal stability can be enhanced by establishing covalent bonds between macrosurfactants and WPUA.

## 4. Conclusions

Because of the surface tensions of PDM-*b*-PRMA is lower than that of PDM-*co*-PBMA (37.36 mN/m), in our research, the CCTP process was chosen to fabricate cationic polymerizable PDM-*b*-PRMA macrosurfactants. A series of PDM-*b*-PRMAs with different HSC lengths were synthesized and used to stabilize WPUA nanoemulsions. The properties of macrosurfactants and the characteristics of WPUA/PDM-*b*-PRMA nanoemulsions were investigated. With the help of the CCTP process, the structure of -C=C was introduced into PDM-*b*-PRMA successfully which may increase the stability of WPUA nanoemulsions due to the chemical interaction. Among the PDM-*b*-PRMAs, when the HSC length was 4, PDM-*b*-PBMA had a more uniform particle size distribution (105.4 nm), good stability and higher surface activity (33.27 mN/m). The WPUA/PDM-*b*-PBMA nanoemulsion also had large solid content (40%), more uniform spherical morphology and smaller colloid size (133.7 nm). The cationic polymerizable PDM-*b*-BMA macrosurfactant developed in this work can be further extended into the preparation of other cationic polymer colloids.

## Figures and Tables

**Table 1:** Chemical compositions of PDM-*b*-PRM with different hydrophobic chain lengths.

Polymer	RMA/g	PDM/g	CoBF Dosage	AIBN/g
PDM-*b*-PMMA	10.0 (MMA)	15.7	100 ppm	0.08
PDM-*b*-PEMA	11.4 (EMA)	15.7	100 ppm	0.08
PDM-*b*-PBMA	14.4 (BMA)	15.7	100 ppm	0.08
PDM-*b*-POMA	19.8 (OMA)	15.7	100 ppm	0.08
PDM-*b*-PLMA	25.4 (LMA)	15.7	100 ppm	0.08

**Table 2:** Effect of HSC length on the molecular weight and double bond content of PDM-*b*-PRMA.

Sample	*M_n_*	*M_w_*	PDI	*M_n_*′	*X*	*C*	*D*
PDM	1200	1400	1.18	1400	18.52	87.5	/
PDM-*b*-PMMA	2000	2400	1.21	2400	10.54	84.4	10.00
PDM-*b*-PEMA	2300	2800	1.24	2700	9.28	82.6	12.28
PDM-*b*-PBMA	2600	3500	1.33	3200	7.85	81.1	14.79
PDM-*b*-POMA	3400	4900	1.42	4900	5.17	69.5	17.68
PDM-*b*-PLMA	4200	7000	1.67	8900	2.85	47.3	22.05
PDM-*co*-PBMA	12,500	23,900	1.93	/	/	/	158.45

**Table 3:** Gas–liquid interface adsorption parameters of PDM-*b*-PRMA and PDM-*co*-PBMA.

Sample	CMC (g/L)	γ_CMC_ (mN/m)	*Γ* (mol/m^2^)	*A* (nm^2^)
PDM-*co*-PBMA	0.16	37.36	0.63 × 10^−6^	2.62
PDM-*b*-PMMA	0.31	35.44	1.15 × 10^−6^	1.44
PDM-*b*-PEMA	0.26	35.14	1.36 × 10^−6^	1.21
PDM-*b*-PBMA	0.33	33.27	2.43 × 10^−6^	0.68
PDM-*b*-POMA	0.22	33.71	1.96 × 10^−6^	0.85
PDM-*b*-PLMA	0.19	34.22	1.62 × 10^−6^	1.03

γ_CMC_ represents surface tension.

**Table 4:** The maximum solid content of WPUA/PDM-*b*-PRMA and WPUA/PDM-*co*-PBMA emulsions.

Sample	The Water Content at PIP	Maximum Solid Content
WPUA/PDM-*b*-PMMA	60%	30%
WPUA/PDM-*b*-PEMA	55%	35%
WPUA/PDM-*b*-PBMA	43%	40%
WPUA/PDM-*b*-POMA	50%	35%
WPUA/PDM-*b*-PLMA	55%	35%
WPUA/PDM-*co*-PBMA	68%	25%

**Table 5:** The surface roughness of WPUA/PDM-*b*-PBMA and WPUA/PDM-*co*-PBMA films.

Sample	Ra_0_/nm	Ra/nm
WPUA/PDM-*b*-PBMA	1.603	1.497
WPUA/PDM-*co*-PBMA	1.542	1.164

Ra_0_ is the average surface roughness at o h and Ra is the average surface roughness after 24 h.
